# A facility and community-based assessment of scabies in rural Malawi

**DOI:** 10.1371/journal.pntd.0009386

**Published:** 2021-06-01

**Authors:** Cristina Galván-Casas, Oriol Mitjá, Sara Esteban, Jacob Kafulafula, Texon Phiri, Íñigo Navarro-Fernández, Concepción Román-Curto, Hassani Mtenje, Gerald Thauzeni, Elizabeth Harawa, Stephano Kaluzi, Mphatso Diere, Mary Mkandawire, Shaibu Malizani, Alex Chifundo, Marta Utrera-Busquets, Mónica Roncero-Riesco, Sara López Martín-Prieto, Iosune Vilanova-Urdániz, Gisela H. Petiti, María Victoria de Gálvez Aranda, Nuria No Pérez, María Rueda Gómez-Calcerrada, Pilar Iranzo, Pilar Escalonilla García-Patos, Magdalena de Troya-Martín, Javier Romero Gomez, Esther Cardeñoso-Alvarez, Sofia Lucas Truyols, Libe Aspe Unanue, Cristina Bajo del Pozo, Alicia Comunión Artieda, Maria Isabel Martínez González, Omar F. López-López, Esther Moreno-Artero, Xavier Cubiró, Iago Meilán-Sánchez, Alejandra Tomás-Velázquez, Cristina López-Sánchez, Eva M. Sánchez-Martínez, Harrison A. Edwards, Maria Herrera Morueco, Julia Zehe Rubiera, Laura Salguero Caldera, Urbano Blanes-Moreno, Maria Uribarren-Movilla, Michael Marks

**Affiliations:** 1 Faculty of Medicine and Health Sciences, Universitat de Barcelona, Barcelona, Spain; 2 Department of Dermatology, Hospital Universitario de Mostoles, University Rey Juan Carlos, Madrid, Spain; 3 DerMalawi Project, Nkhotakota, Malawi; 4 Fight AIDS and Infectious Diseases Foundation, Badalona, Spain; 5 Hospital Universitari Germans Trias i Pujol, Badalona, Spain; 6 Lihir Medical Centre-International SOS, Londolovit, Lihir Island, Papua New Guinea; 7 Department of Dermatology, Hospital Universitario de Cruces, Bilbao, Spain; 8 Nkhotakota District Hospital, Nkhotakota, Malawi; 9 Alinafe Health Center, Njewa, Malawi; 10 Department of Dermatology, Hospital Universitario de Cabueñes, Gijón, Spain; 11 Department of Dermatology, Complejo Asistencial Universitario de Salamanca, Salamanca, Spain; 12 Mwansambo Health Center, Mwansambo, Malawi; 13 Kapiri Health Center, Kapiri, Malawi; 14 Benga Health Center Benga, Malawi; 15 Thavite Health Center, Thavite, Malawi; 16 Department of Dermatology, Hospital General La Mancha Centro, Ciudad Real, Spain; 17 Consulta privada, Seville, Spain; 18 Department of Dermatology, Hospital General Universitario de Elche, Alicante, Spain; 19 Department of Dermatology, Hospital Mòisses Broggi, Barcelona, Spain; 20 Faculty of Medicine, Universidad de Málaga, Málaga, Spain; 21 Department of Dermatology, Complejo Hospitalario Universitario de Vigo, Vigo, Spain; 22 Department of Dermatology, Hospital Viamed Virgen de la Paloma, Madrid, Spain; 23 Department of Dermatology, Hospital Clínic, Barcelona, Spain; 24 Department of Dermatology, Complejo Asistencial de Ávila, Ávila, Spain; 25 Department of Dermatology, Hospital Costa del Sol, Marbella, Spain; 26 Department of Dermatology, Hospital Quironsalud, Málaga, Spain; 27 Department of Dermatology, Consorci Hospital General Universitari, València, Spain; 28 Department of Dermatology, Hospital Universitario Araba, Vitoria-Gasteiz, Spain; 29 Department of Dermatology, Complejo Hospitalario de Palencia, Palencia, Spain; 30 Department of Dermatology, Centro de Diagnóstico Médico, Ayuntamiento de Madrid, Madrid, Spain; 31 Consulta privada, A Coruña, Spain; 32 Department of Dermatology, Clínica Universidad de Navarra, Madrid, Spain; 33 Department of Dermatology, Hospital de la Santa Creu i Sant Pau, Barcelona, Spain; 34 Department of Dermatology, Complexo Hospitalario Universitario A Coruña, A Coruña, Spain; 35 Department of Dermatology, Clínica Universidad de Navarra, Pamplona, Spain; 36 Department of Dermatology, Hospital Universitario Doctor Peset, Valencia, Spain; 37 Department of Dermatology, Princess Alexandra Hospital, Brisbane, Australia; 38 School of Public Health, University of Queensland, Brisbane, Australia; 39 Dermatology Research Centre, University of Queensland, Diamantina Institute, Brisbane, Australia; 40 Department of Internal Medicine, Hospital Universitario Infanta Leonor, Madrid, Spain; 41 Hospital Plat, Barcelona, Spain; 42 Hospital Universitario Germans Trias y Pujol, Barcelona, Spain; 43 Francisco de Vitoria University, Madrid, Spain; 44 Alcalá University, Madrid, Spain; 45 Clinical Research Department, Faculty of Infectious and Tropical Diseases, London School of Hygiene & Tropical Medicine, London, United Kingdom; Brighton and Sussex University Hospitals NHS Trust, UNITED KINGDOM

## Abstract

**Background:**

Scabies is a neglected tropical disease of the skin, causing severe itching, stigmatizing skin lesions and systemic complications. Since 2015, the DerMalawi project provide an integrated skin diseases clinics and Tele-dermatology care in Malawi. Clinic based data suggested a progressive increase in scabies cases observed. To better identify and treat individuals with scabies in the region, we shifted from a clinic-based model to a community based outreach programme.

**Methodology/Principal findings:**

From May 2015, DerMalawi project provide integrated skin diseases and Tele-dermatological care in the Nkhotakota and Salima health districts in Malawi. Demographic and clinical data of all patients personally attended are recorded. Due to a progressive increase in the number of cases of scabies the project shifted to a community-based outreach programme. For the community outreach activities, we conducted three visits between 2018 to 2019 and undertook screening in schools and villages of Alinafe Hospital catchment area. Treatment was offered for all the cases and school or household contacts. Scabies increased from 2.9% to 39.2% of all cases seen by the DerMalawi project at clinics between 2015 to 2018. During the community-based activities approximately 50% of the population was assessed in each of three visits. The prevalence of scabies was similar in the first two rounds, 15.4% (2392) at the first visit and 17.2% at the second visit. The prevalence of scabies appeared to be lower (2.4%) at the third visit. The prevalence of impetigo appeared unchanged and was 6.7% at the first visit and 5.2% at the final visit.

**Conclusions/Significance:**

Prevalence of scabies in our setting was very high suggesting that scabies is a major public health problem in parts of Malawi. Further work is required to more accurately assess the burden of disease and develop appropriate public health strategies for its control.

## Author summary

Scabies is an infestation of the skin caused by a mite. There is limited data on how common scabies is in sub-Saharan Africa, including Malawi. The DerMalawi project has been providing care for dermatological conditions in rural Malawi since 2015. Between 2015 and 2018 we observed an increase in patients with scabies attending for treatment. In response the project shifted from providing care at clinics to an approach using community-based outreach. Between 2018 and 2019 we conducted community-based activities on three occasions in an area of approximately 30,000 individuals. The DerMalawi team visited schools and villages to identify and treat cases of scabies and their contacts. We were able to examine about 50% of the population on each visit. Initially a large proportion of the population had scabies (15%) and this was similar during our second community survey. At our third survey this appeared to have decreased to 2% but it is difficult for us to know if this is because of treatment given in the previous rounds. Scabies is a major problem in rural populations in Malawi and public health strategies are needed to reduce the disease burden.

## Introduction

Scabies is a skin disease caused by infestation with the mite, *Sarcoptes scabiei var*. *Hominis*. Human transmission is predominantly via skin-to-skin contact. Around 200–300 million people are affected each year [1, 2], particularly among poor populations living in crowded conditions in tropical areas. Until recently there has been limited data on the epidemiology of scabies in sub-Saharan Africa and in particular limited population level estimates of disease prevalence[3]. More recent data from Ethiopia, Liberia and Ghana all suggest that scabies is a major public health problem in sub-Saharan Africa[4–6].

Permethrin 5% topical ointment is widely considered the first line treatment for treatment of individuals with scabies[7] but there is increasing evidence that community wide treatment with ivermectin is the best strategy for control in highly endemic areas. The major challenge to the use of ivermectin is the current recommendation for two separated doses and its relative contraindication in pregnant and breastfeeding women and children under five years of age. [8–13].

Standard guidelines for scabies control are based on treatment of cases and contacts [14], but this approach is not effective in highly-endemic areas [15–17]. Different mass treatment approaches have resulted in positive outcomes [18–20] Mass drug administration of ivermectin as part of lymphatic filariasis control in Zanzibar was associated with a 68% decrease of scabies treatment’s prescription[21]. The first comparative trial of mass drug administration for scabies was the Skin Health Intervention Fiji Trial (SHIFT) which found that ivermectin-based mass drug administration decreased the prevalence of scabies by 94% 1 year after the intervention, and was superior to both mass drug administration of topical permethrin and standard care[9].

Better data is needed on the epidemiology of scabies in sub-Saharan Africa. We report facility and community based data on scabies in rural Malawi, collected as part of the DerMalawi project.

## Methods

### Ethics statement

The findings reported in this paper reflect data collected during routine clinical health work, while the patients’ screening and treatment was organized in local schools or villages as opposed to distant clinics. Routine clinical health work does not require IRB approval. These arrangements were approved by the Malawi health authorities (Nkhotakota District Health Officer) who requested the clinical health work which is the base of the study and agreed with the study related data collection and handling. All participants provided oral informed consent in the presence of witnesses and in their mother language (Chichewa).

### The DerMalawi project

**Fig 1:**
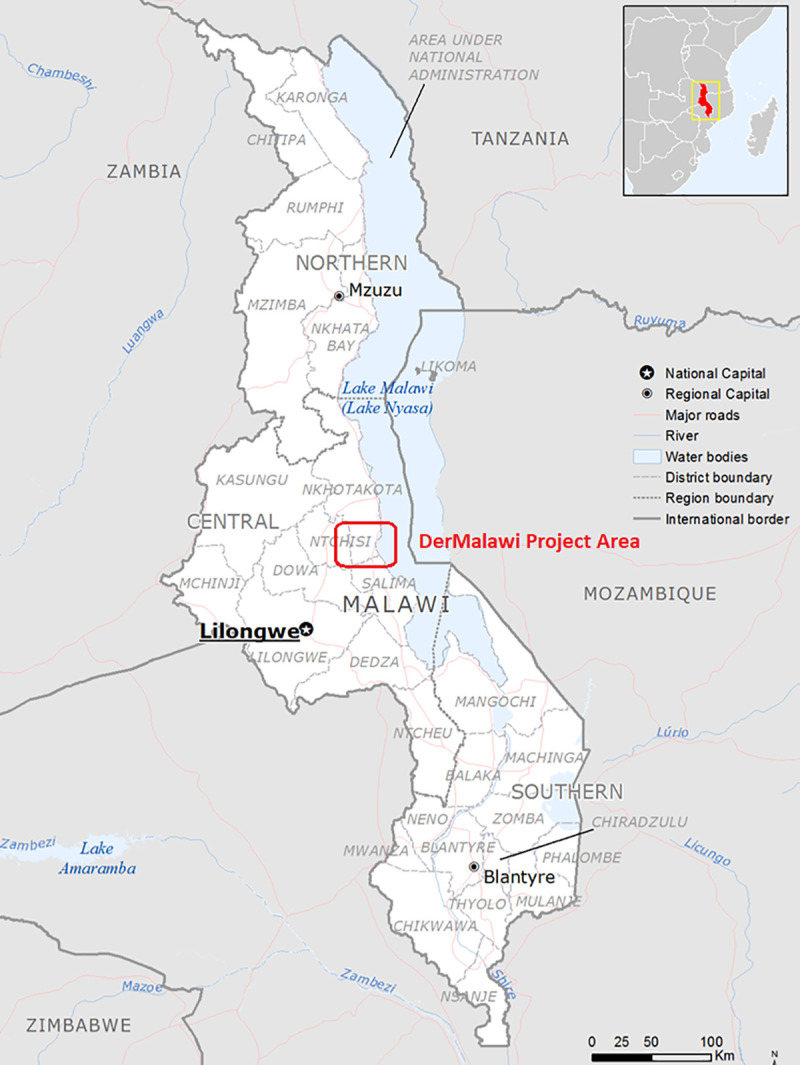
Map of Malawi indicating the location of the DerMalawi Project (Base layer: https://commons.wikimedia.org/wiki/File:Malawi_Base_Map.png).

**Fig 2:**
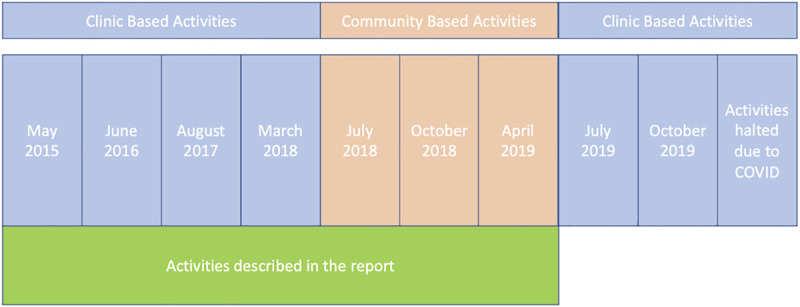
Timings of activities conducted by the DerMalawi Project 2015–2020.

There is limited access to dermatology in Malawi with only two trained dermatologists in the country. The DerMalawi project (http://www.dermalawi.com/) is an ongoing project in collaboration with the Malawian Health authorities that was established in May 2015 to provide integrated skin diseases care and Tele-dermatological consultations to communities in the Nkhotakota and Salima health districts in Malawi (Fig 1). The project is governed by a memorandum of understanding between a group of Spanish dermatologists and the Malawian Health authorities. As part of the project Dermatologists are approved to work in the area from the Malawi Medical Council and local oversite for clinical work is provided by the Nkhotakota (Malawi) District Medical Officer. The Benga Parish Missionary Community San Paul de Apostle (MCSPA) acts as institutional liaison in Malawi between the project partners. The DerMalawi team comprises Spanish dermatologists, staff from Alinafe Hospital, trained Chichewa-English translators and community health manager. We report here on activities conducted between 2015–2019 (Fig 2).

### Clinic based data collection

Since the beginning of the DerMalawi project individuals with any skin problem were invited to attend for facility-based skin screening. As part of these activities the demographic and clinical data of all patients are recorded in spreadsheet, using tablets. Among the clinical data we collected personal and family history, symptoms, dermatological examination, complementary examinations performed, coded diagnosis (ICD-10) and prescribed and administered treatment. This data included basic information on the number of individuals assessed and the number of cases of major common skin conditions based on clinical diagnosis. Between May 2015 to March 2018, clinic-based data suggested that there had been a progressive increase in the proportion and absolute number of cases of scabies seeking care. In response, the Malawi Health authorities requested the DerMalawi project to shift from a clinic-based model to a community-based outreach programme to try and better identify and treat individuals with scabies in the region. This screening and treatment strategy was developed between the DerMalawi project and the Malawian Health and Educational Authorities.

### Community based data collection

Community outreach activities took place in the Alinafe Health Center catchment area, Nkhotakota and Salima districts, Malawi, which has a population of approximately 30,000 individuals. Three rounds of community dermatology outreach activities were conducted between July 2018 and April 2019 in both schools and communities.

A week before each round we conducted health awareness at community meetings, school meetings and through public notices, pamphlets and posters in cooperation with village leaders. These activities were conducted by Malawian health staff in collaboration with the DerMalawi project and explained the background and purpose of the community-based activities. The DerMalawi team visited schools class by class and in villages attempted to go house by house trying to reach all individuals present at the school or at home. All individuals were invited for skin screening regardless of whether they reported itch of a skin problem. A private area was setup for screening at each house or school visited. As with clinic-based data collection we collected information on the number of individuals seen and demographics but focused clinical examination on scabies and impetigo in view of the marked increase we had seen at the health facility.

### Examination

In both clinic and community settings a dermatologist carried out a standardized skin examination. Scabies was classified on the basis of a clinical diagnosis by a trained dermatologist. Dermatoscopy was used in cases of uncertainty. Impetigo was defined on the basis of a clinical examination without microbiological confirmation based on the presence of pustules, sores and adherent golden crusts.

### Treatment

All individuals diagnosed with scabies were offered two doses of oral ivermectin (200 μg per kilogram of body weight) or topical permethrin cream where ivermectin was contra-indicated (pregnancy, breastfeeding, weight under 15kg). The first dose was given under direct supervision. The team returned after 7–14 days to provide a second dose. We attempted to provide treatment to all class-mates where cases of scabies were found during school visits and to the household of cases of found during either school or household visits. Medication was provided by the DerMalawi project with approval from the Malawian Health Authorities.

### Analysis

The DerMalawi project area has 14 schools, 94 villages and five health facilities. For the purpose of project implementation, we grouped schools and their surrounding villages into groups and visited each in turn. A school and its linked villages were considered as a single school-catchment area for the purpose of analysis. For clinic based data we report the absolute number of cases of scabies and the proportion of all skin problems seen that were due to scabies. For the community data we report the prevalence of scabies (with and without secondary bacterial infection), for each school catchment area, and the overall DerMalawi project area.

## Results

### DerMalwi project acitivites

The population living in the catchment area of the Alinafe Health Centre is estimated at 29,780 people (2018 census). Between 2015 and 2019 the DerMalawi Project conducted 4 health centre based clinics and three community based skin screening activities.

### Facility based activities

A total of 4,769 people were assessed as part of facility-based skin screening. The proportion and absolute number of scabies diagnosed increased from 13 (2.8%) individuals in 2015 to 792 (38.7%) individuals in 2018. There appeared to be decline in the number of cases of fungal infections seen but no significant changes in the proportions of any other key diseases (Table 1).

**Table 1:** Cases seen during clinic-based activities.

Diagnosis	May 2015	June2016	August 2017	August 2018
**Scabies**	13 (2.9%)	9 (1.2%)	146 (15.2%)	792 (39.2%)
**Leprosy**	5 (1.1%)	6 (0.8%)	10 (1.0%)	4 (0.2%)
**Impetigo**	18 (3.9%)	24 (3.1%)	27 (2.8%)	46(2.3%)
**Skin ulcer**	3 (0.7%)	4(0.5%)	8 (0.8%)	30 (1.5%)
**Skin Cancer**	16 (3.5%)	28 (3.6%)	40 (4.2%)	33 (1.6%)
**Fungal disease**	192 (42.1%)	243(31.3%)	334 (34.8%)	536 (26.6%)
**Total**	456	776	960	2018

### Community based activities

A total of 15,487 (52% of catchment are population) were screened in July 2018, followed by 16,619 (55.8%) in October 2018 and 12,617 (42.4%) in April 2019 (Table 2). The prevalence of scabies was similar during our first two community visits; 15.4% (2392) in July 2018 and 17.2% (2855) in October 2018 but only 2.4% in April 2019. All individuals diagnosed with scabies received treatment with IVM but only 47% of cases seen in the first community visit and only 55% of cases seen during the second community visit received an observed second dose of treatment. As well as index cases, 5,204 individuals and 7,888 individuals received treatment as contacts during these visits.

**Table 2:** Individuals seen during community based activities.

		July 2018 (N = 15487)	October 2018 (N = 16619)	April 2019 (N = 12617)
**Age Group**	0-4yrs	2384 (15.4%)	1894 (11.4%)	2143 (17.0%)
	5-9yrs	3411 (22.0%)	4242 (25.5%)	2626 (20.8%)
	10-14yrs	3994 (25.8%)	5490 (33.0%)	2933 (23.2%)
	15-24yrs	2402 (15.5%)	2259 (13.6%)	2033 (16.1%)
	24-34yrs	1344 (8.7%)	1106 (6.7%)	1120 (8.9%)
	> = 35yrs	1952 (12.6%)	1628 (9.8%)	1762 (14.0%)
**Gender**	Female	8714 (56.3%)	9152 (55.1%)	7246 (57.4%)
	Male	6773 (43.7%)	7467 (44.9%)	5371 (42.6%)
**Scabies**		2392 (15.4% - 95%CI 14.9–16.0%)	2855 (17.2% - 95% CI 16.6–17.8%)	303 (2.4% - 95% CI 2.1–2.7%)
**Impetigo**		1032 (6.7% - 95%CI 6.3–7.1%)	704 (4.2% - 95%CI 3.9–4.6%)	658 (5.2% - 95% CI 4.8–5.6%)

**Fig 3:**
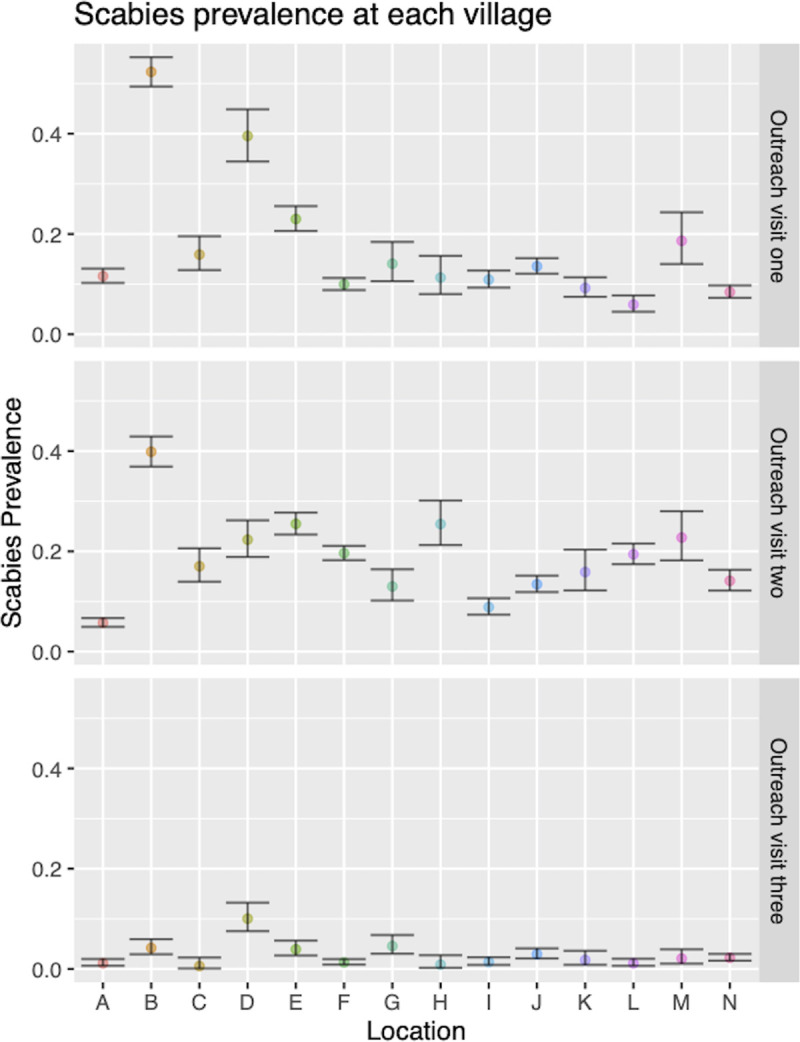
Scabies prevalence by village at each of the three community outreach events conducted by the DerMalawi Project.

At each community visit the prevalence of scabies varied markedly between villages (range 5–40%, Fig 3). The prevalence of scabies was highest amongst children under 5 (Table 3) at each visit. No cases of crusted scabies were found. The prevalence of impetigo was 6.7% (1032), 4.2% (704), and 5.2% (658) across the three visits.

**Table 3:** Prevalence of scabies and impetigo by age group during each visit.

	Visit 1		Visit 2		VISIT 3	
Age Group	Prevalence of Scabies % (95% CI)	Prevalence of Impetigo % (95% CI)	Prevalence of Scabies % (95% CI)	Prevalence of Impetigo % (95% CI)	Prevalence of Scabies% (95% CI)	Prevalence of Impetigo
0–4	20.6% (19–22.2%)	10.5% (9.3–11.8%)	22.3% (20.4–24.2%)	5.5% (4.6–6.7%)	4% (3.2–4.9%)	6% (5.1–7.1%)
05–10	16% (14.8–17.3%)	7.9% (7–8.9%)	18.2% (17–19.4%)	5.2% (4.5–5.9%)	3.2% (2.6–4%)	9.1% (8–10.3%)
10–14	15.8% (14.7–17%)	7.2% (6.4–8%)	18.7% (17.7–19.8%)	4.7% (4.1–5.3%)	2.8% (2.2–3.5%)	7% (6.1–8%)
15–24	14.7% (13.3–16.1%)	5.2% (4.3–6.1%)	15.2% (13.8–16.8%)	3.3% (2.6–4.1%)	1.3% (0.9–1.9%)	2.9% (2.2–3.7%)
25–34	13.7% (11.9–15.7%)	3.9% (3–5.2%)	11.2% (9.4–13.3%)	1.7% (1.1–2.7%)	1% (0.5–1.8%)	1.1% (0.6–1.9%)
≥35	9.7% (8.4–11.1%)	2.5% (1.9–3.3%)	10.3% (8.9–11.9%)	1.8% (1.3–2.7%)	0.9% (0.5–1.4%)	0.8% (0.5–1.4%)

## Discussion

There is limited data on the prevalence of scabies in much of sub-Saharan Africa although several recent studies suggest that it is common[22]. There have been large scale outbreaks of scabies reported from Ethiopia[4, 22] in the context of drought, and reports from both rural and peri-urban regions of West Africa demonstrating high prevalences of scabies[5, 6, 23]. Prior to the DerMalawi project there has been no recent data on scabies in Malawi. The data from the DerMalawi project suggests that scabies is a major problem in the area with especially high prevalences amongst children. In keeping with data from other parts of sub-Saharan Africa we did not notice a particularly high burden of impetigo compared to that reported in the Pacific region[24, 25].

The DerMalawi project has been working in Malawi since 2015. Over that time we noted a marked increase in presentations to our service for scabies. When we switched to a community rather than a clinic based model we found an extremely high community prevalence of scabies of 15%. Over the course of three community outreach activities the prevalence appeared to decline markedly but whether this reduction is directly related to our activities and treatment is unclear.

There was no change in scabies prevalence between July 2018 and October 2018 suggesting that at the coverage achieved the treatment of cases and a proportion of their contacts was inadequate to affect scabies prevalence. This lack of change is likely due to low population coverage. We did not record when people declined to be examined but based on the number of people seen and the known population of the Alinafe catchment area, when we switched from a clinic to a community based strategy we still reached only about 50% of the population. Such low coverage invariably leads to a large number of untreated scabies cases that might act as a reservoir for infection. In addition, most individuals received only a single observed dose of ivermectin limiting the efficacy of treatment for individual patients. All three visits were conducted during the dry season, so differences in prevalence seem unlikely to be related to seasonality[23]. However given the before and after nature of the data we can not exclude other secular changes in scabies prevalence, which have been observed in some settings. Unlike research interventions that have been carried out in islands or confined populations and have maintained the effects for years [17], the activities reported here were carried out as part of a long term dermatological assistance programme and the patients in our target area, are surrounded by neighbouring communities with potentially high scabies rates. These surrounding areas therefore serve as a further reservoir from which reintroduction can occur. Strategies to improve programme coverage including more extended community outreach and potentially implementation on a larger scale, to avoid inward transmission from outside the area, should be considered as possible strategies.

The markedly reduced prevalence observed in our visit in April 2019 despite low treatment coverage, and in the absence of any effect seen previously, suggests that the observed reduction is unlikely to be due to treatment and more likely reflects changes in the population assessed at each time point. In keeping with this there were differences in both the demographic structure of the population seen in our final community visit and in the number of people seen at each screening site across each of our visits. We noted that in the final visit a higher proportion of individuals declined examination particularly in the absence of self-reported itch. For these reasons it seems likely that the reduction seen is most likely a reflection of sampling bias not a true difference.

Scabies remains as an unresolved matter for global health, continues to be widespread and is responsible for intense suffering, health and social complications. New drugs, dissemination models and treatment and preventive strategies are needed to achieve better results. Targets for the establishment of scabies control programmes have been included in the new WHO NTD Roadmap 2021–2030 and our data suggests such a programme should be considered in Malawi alongside other national NTD programmes.

## Supporting information

S1 Project Dataset Patients attended; Scabies, Impetigo and demographic data. (XLSX)

## References

[1] GBD 2015 Disease and Injury Incidence and Prevalence Collaborators. Global, regional, and national incidence, prevalence, and years lived with disability for 310 diseases and injuries, 1990–2015: a systematic analysis for the Global Burden of Disease Study 2015. Lancet Lond Engl. 2016;388: 1545–1602. doi: 10.1016/S0140-6736(16)31678-6 27733282 PMC5055577

[2] Chosidow O. Clinical practices. Scabies. N Engl J Med. 2006;354: 1718–1727. doi: 10.1056/NEJMcp052784 16625010

[3] Engelman D, Steer AC. Control Strategies for Scabies. Trop Med Infect Dis. 2018;3. doi: 10.3390/tropicalmed3030098 30274494 PMC6160909

[4] Enbiale W, Ayalew A. Investigation of a Scabies Outbreak in Drought-Affected Areas in Ethiopia. Trop Med Infect Dis. 2018;3: 114. doi: 10.3390/tropicalmed3040114 30380650 PMC6306922

[5] Collinson S, Timothy J, Zayzay SK, Kollie KK, Lebas E, Candy N, et al. The prevalence of scabies in Monrovia, Liberia: A population-based survey. PLoS Negl Trop Dis. 2020;14: e0008943. doi: 10.1371/journal.pntd.0008943 33284821 PMC7746289

[6] Boateng LA, Partnership TGSSR, Adom F, Angwaawie P, Boateng L, Danquah E, et al. Healthcare-seeking behaviour in reporting of scabies and skin infections in Ghana: A review of reported cases. Trans R Soc Trop Med Hyg. [cited 31 Aug 2020]. doi: 10.1093/trstmh/traa071 32853365

[7] Usha V, Gopalakrishnan Nair TV. A comparative study of oral ivermectin and topical permethrin cream in the treatment of scabies. J Am Acad Dermatol. 2000;42: 236–240. doi: 10.1016/S0190-9622(00)90131-2 10642678

[8] Wong L-C, Amega B, Barker R, Connors C, Dulla ME, Ninnal A, et al. Factors supporting sustainability of a community-based scabies control program. Australas J Dermatol. 2002;43: 274–277. doi: 10.1046/j.1440-0960.2002.00626.x 12423434

[9] Romani L, Whitfeld MJ, Koroivueta J, Kama M, Wand H, Tikoduadua L, et al. Mass Drug Administration for Scabies Control in a Population with Endemic Disease. N Engl J Med. 2015;373: 2305–2313. doi: 10.1056/NEJMoa1500987 26650152

[10] Ly F, Caumes E, Ndaw CAT, Ndiaye B, Mahé A. Ivermectin versus benzyl benzoate applied once or twice to treat human scabies in Dakar, Senegal: a randomized controlled trial. Bull World Health Organ. 2009;87: 424–430. doi: 10.2471/blt.08.052308 19565120 PMC2686207

[11] Hay RJ, Steer AC, Chosidow O, Currie BJ. Scabies: a suitable case for a global control initiative. Curr Opin Infect Dis. 2013;26: 107–109. doi: 10.1097/QCO.0b013e32835e085b 23302759

[12] Engelman D, Kiang K, Chosidow O, McCarthy J, Fuller C, Lammie P, et al. Toward the global control of human scabies: introducing the International Alliance for the Control of Scabies. PLoS Negl Trop Dis. 2013;7: e2167. doi: 10.1371/journal.pntd.0002167 23951369 PMC3738445

[13] Currie BJ, McCarthy JS. Permethrin and ivermectin for scabies. N Engl J Med. 2010;362: 717–725. doi: 10.1056/NEJMct0910329 20181973

[14] Mounsey KE, McCarthy JS. Treatment and control of scabies. Curr Opin Infect Dis. 2013;26: 133–139. doi: 10.1097/QCO.0b013e32835e1d57 23438966

[15] La Vincente S, Kearns T, Connors C, Cameron S, Carapetis J, Andrews R. Community management of endemic scabies in remote aboriginal communities of northern Australia: low treatment uptake and high ongoing acquisition. PLoS Negl Trop Dis. 2009;3: e444. doi: 10.1371/journal.pntd.0000444 19478832 PMC2680947

[16] Lawrence G, Leafasia J, Sheridan J, Hills S, Wate J, Wate C, et al. Control of scabies, skin sores and haematuria in children in the Solomon Islands: another role for ivermectin. Bull World Health Organ. 2005;83: 34–42. doi: /S0042-96862005000100012 15682247 PMC2623469

[17] Marks M, Romani L, Sokana O, Neko L, Harrington R, Nasi T, et al. Prevalence of scabies and impetigo three years after mass drug administration with ivermectin and azithromycin. Clin Infect Dis Off Publ Infect Dis Soc Am. 2019. doi: 10.1093/cid/ciz444 31131410 PMC7145994

[18] Kearns TM, Speare R, Cheng AC, McCarthy J, Carapetis JR, Holt DC, et al. Impact of an Ivermectin Mass Drug Administration on Scabies Prevalence in a Remote Australian Aboriginal Community. PLoS Negl Trop Dis. 2015;9: e0004151. doi: 10.1371/journal.pntd.0004151 26516764 PMC4627839

[19] Taplin D, Porcelain SL, Meinking TL, Athey RL, Chen JA, Castillero PM, et al. Community control of scabies: a model based on use of permethrin cream. Lancet Lond Engl. 1991;337: 1016–1018. doi: 10.1016/0140-6736(91)92669-s 1673175

[20] Evans DS, Alphonsus K, Umaru J, Eigege A, Miri E, Mafuyai H, et al. Status of Onchocerciasis transmission after more than a decade of mass drug administration for onchocerciasis and lymphatic filariasis elimination in central Nigeria: challenges in coordinating the stop MDA decision. PLoS Negl Trop Dis. 2014;8: e3113. doi: 10.1371/journal.pntd.0003113 25233351 PMC4169246

[21] Mohammed KA, Deb RM, Stanton MC, Molyneux DH. Soil transmitted helminths and scabies in Zanzibar, Tanzania following mass drug administration for lymphatic filariasis—a rapid assessment methodology to assess impact. Parasit Vectors. 2012;5: 299. doi: 10.1186/1756-3305-5-299 23259465 PMC3543323

[22] Azene AG, Aragaw AM, Wassie GT. Prevalence and associated factors of scabies in Ethiopia: systematic review and Meta-analysis. BMC Infect Dis. 2020;20. doi: 10.1186/s12879-020-05106-3 32460770 PMC7254678

[23] Armitage EP, Senghore E, Darboe S, Barry M, Camara J, Bah S, et al. High burden and seasonal variation of paediatric scabies and pyoderma prevalence in The Gambia: A cross-sectional study. Taylan Ozkan A, editor. PLoS Negl Trop Dis. 2019;13: e0007801. doi: 10.1371/journal.pntd.0007801 31609963 PMC6812840

[24] Mason DS, Marks M, Sokana O, Solomon AW, Mabey DC, Romani L, et al. The Prevalence of Scabies and Impetigo in the Solomon Islands: A Population-Based Survey. Vinetz JM, editor. PLoS Negl Trop Dis. 2016;10: e0004803. doi: 10.1371/journal.pntd.0004803 27348119 PMC4922659

[25] Tikoduadua L, Kama M, Romani L, Steer AC, Tuicakau M, Whitfeld MJ, et al. The Epidemiology of Scabies and Impetigo in Relation to Demographic and Residential Characteristics: Baseline Findings from the Skin Health Intervention Fiji Trial. Am J Trop Med Hyg. 2017;97: 845–850. doi: 10.4269/ajtmh.16-0753 28722612 PMC5590570

